# Supplementary material for: Identification of photoperiod-induced specific miRNAs in the adrenal glands of Sunite sheep (Ovis aries)
Source: Front Vet Sci. 2022 Jul 22;9:888207. doi: 10.3389/fvets.2022.888207 (PMC9354845; doi:10.3389/fvets.2022.888207)
Supplement: Supplementary file 1 [file Data_Sheet_1.ZIP › Supplementary file/miRDeep2 file.zip!/miRDeep2 file/Know_Structure/LP42A3/oar-let-7d.pdf]

miRBase precursor	: oar-let-7d
Total read count	: 19617
oar-let-7d read count	: 18133
remaining reads	: 1484

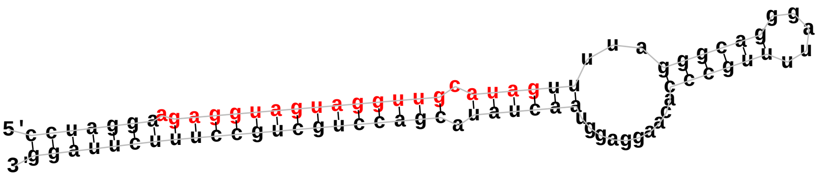

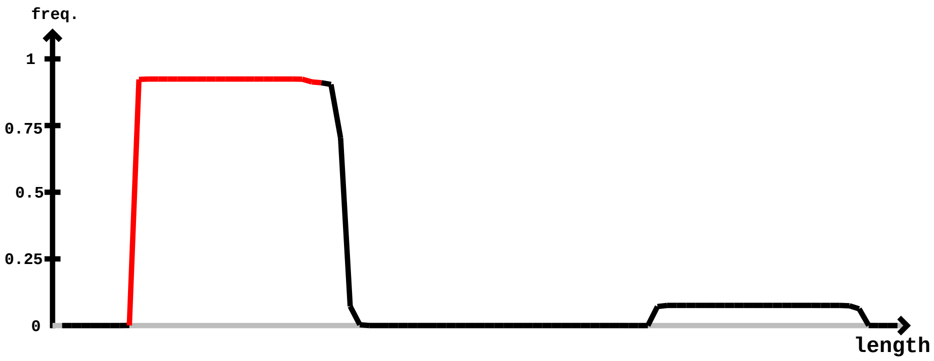


```
        oar-let-7d
5'- ccuaggaagagguaguagguugcauaguuuuagggcagggauuuugcccacaaggagguaacuauacgaccugcugccuuucuuagg  -3'   exp
    (((((((.((((((((((((((.((((((...((((((.....))))))..........)))))).)))))))))))))))))))))   reads   mm   sample
    ......aagagguaguagguugcau..............................................................   1       0    seq
    ......aagagguaguagguugcauagu...........................................................   5       0    seq
    ......aagagguaguagguugcauaguu..........................................................   4       0    seq
    .......agagguaguagguugca...............................................................   8       0    seq
    .......agagguaguagguuAcau..............................................................   1       1    seq
    .......agagguaguagguugcau..............................................................   201     0    seq
    .......agagguaguagguugcaua.............................................................   60      0    seq
    .......agagguaguagguugcauag............................................................   123     0    seq
    .......agagguaguagguugcauagu...........................................................   3935    0    seq
    .......agagguaguagguugcauaguu..........................................................   12354   0    seq
    .......agagguaguagguugcauaguuu.........................................................   1355    0    seq
    .......agagguaguagguugcauaguuuu........................................................   60      0    seq
    ........gagguaguagguugcauag............................................................   1       0    seq
    ........gagguaguagguugcauagu...........................................................   5       0    seq
    ........gagguaguagguugcauaguu..........................................................   19      0    seq
    ........gagguaguagguugcauaguuu.........................................................   1       0    seq
    .............................uuagggcagggauuuugcccacaaggagg.............................   1       0    seq
    .............................................................cuauacgaccugcugcU.........   1       1    seq
    .............................................................cuauacgaccugcugccuu.......   1       0    seq
    .............................................................cuauacgaccugcugccuuu......   23      0    seq
    .............................................................cuauacgaccugcugccuuuc.....   202     0    seq
    .............................................................cuauacgaccugcugccuuucu....   1168    0    seq
    .............................................................cuauacgaccugcugccuuucuu...   4       0    seq
    ..............................................................uauacgaccugcugccuuu......   5       0    seq
    ..............................................................uauacgaccugcugccuuuc.....   19      0    seq
    ..............................................................uauacgaccugcugccuuucu....   57      0    seq
    ..............................................................uauacgaccugcugccuuucuu...   2       0    seq
    ...............................................................auacgaccugcugccuuucu....   1       0    seq
```
